# In Situ Synthesis of a Magnetic Graphene Platform for the Extraction of Benzimidazoles from Food Samples and Analysis by High-Performance Liquid Chromatography

**DOI:** 10.1155/2017/3018198

**Published:** 2017-05-04

**Authors:** Qianchun Zhang, Yulan Liu, Xingyi Wang, Huimin Li, Junyu Chen

**Affiliations:** School of Biology and Chemistry, Xingyi Normal University for Nationalities, Xingyi 562400, China

## Abstract

A novel method was proposed for the determination of five benzimidazoles (oxfendazole, mebendazole, flubendazole, albendazole, and fenbendazole) using magnetic graphene (G-Fe_3_O_4_). G-Fe_3_O_4_ was synthesized via in situ chemical coprecipitation. The properties of G-Fe_3_O_4_ were characterized by various instrumental methods. G-Fe_3_O_4_ exhibited a great adsorption ability and good stability towards analytes. Various experimental parameters that might affect the extraction efficiency such as the amount of G-Fe_3_O_4_, extraction solvent, extraction time, and desorption conditions were evaluated. Under the optimized conditions, a method based on G-Fe_3_O_4_ magnetic solid-phase extraction coupled with high-performance liquid chromatography was developed. A good linear response was observed in the concentration range of 0.100–100 *μ*g/L for the five benzimidazoles, with correlation coefficients ranging from 0.9966 to 0.9998. The limits of detection (*S*/*N* = 3) of the method were between 17.2 and 32.3 ng/L. Trace benzimidazoles in chicken, chicken blood, and chicken liver samples were determined and the concentrations of oxfendazole, mebendazole, flubendazole, and fenbendazole in these samples were 13.0–20.2, 1.62–4.64, 1.94–6.42, and 0.292–1.04 ng/g, respectively. The recovery ranged from 83.0% to 115%, and the relative standard deviations were less than 7.9%. The proposed method was sensitive, reliable, and convenient for the analysis of trace benzimidazoles in food samples.

## 1. Introduction

Benzimidazoles (BMZs) are broad-spectrum anthelmintics and have been used in animal husbandry for the prevention and control of a wide variety of gastrointestinal nematodes in aquaculture, agriculture, and veterinary practices [1–4]. BMZs and their metabolites have been shown to cause teratogenic and embryotoxic effects in some species [5]. BMZs are difficult to degrade due to their high stability and complex molecular structures. As such, they are considered to be very significant food product pollutants. Due to the low concentration of the target compounds and the complex nature of their matrices, trace-level detection and identification as well as efficient sample preparation are critical. Therefore, the determination of BMZs in biological matrices remains a challenge. Towards this end, different preconcentration methods are necessary for the extraction of trace BMZs.

Sample preparation affects nearly all subsequent assay steps and is critical to the unequivocal identification, confirmation, and quantification of analytes [6]. To date, a number of sample preconcentration techniques have been reported for the isolation and enrichment of individual BMZs and their metabolites in food products prior to instrumental analysis. Various groups have reported methods to isolate and enrich BMZs by solid-phase extraction (SPE) [7, 8]; some other methods and sample preconcentration techniques have been developed, including liquid-liquid extraction [9, 10], stir cake sorptive extraction [3], solid-phase microextraction (SPME) [11], ultrasound-assisted cloud-point extraction [12], supercritical fluid extraction [13], ultrasound-assisted emulsification-microextraction [14], QuEChERS [15], and ionic liquid-dispersive liquid-liquid microextraction [16]. However, in order to overcome the extensive time requirement and large volume of samples and toxic organic solvents, a new mode for SPE called magnetic solid-phase extraction (MSPE) was recently developed and has attracted significant attention in bioseparation and chemical analyses [17, 18]. In MSPE, iron oxide materials are typically used as adsorbents and they possess some unique properties such as large surface areas, surface modifiability, excellent superparamagnetic propensity, and great biocompatibility. The development of magnetic solid-phase extraction (MSPE) overcomes the limitations of conventional SPE processes; in comparison with traditional SPE, MSPE is a modern and miniaturized technique which demands small volume of sample and solvents for easy clean-up procedures and also has other advantages, including the large interfacial area for mass transmission, easy operation, minimal time requirement, and amenability towards high throughput measurements [19–22]. The separation process in MSPE can be performed directly from a sample matrix containing a magnetic solid absorbent with the aid of an external magnet, without the need for additional centrifugation or filtration procedures, thus facilitating and simplifying the separation and collection processes. The aforementioned merits render MSPE a promising technique for sample preparation. To date, MSPE has been used in many fields [18, 23–27]. Notably, some research groups have utilized this method for the extraction of BMZs [28, 29].

In order to enhance the selectivity and extraction of MSPE, magnetic particles are usually subjected to surface functionalization with appropriate recognition molecules. Fe_3_O_4_ nanoparticles (NPS) are the most popular particles due to their low cost and toxicity. Fe_3_O_4_ NPS have been modified and functionalized with several different materials. Specifically, various research groups have functionalized the NPS with C_8_ [30], carbon nanotubes [31], polymers [32], and octadecyl moieties [33] and have used the modified Fe_3_O_4_ NPS to extract toxic substances and environmental pollutants from water and food. Among the aforementioned modified materials, graphene is a novel and interesting carbon-based material, it was discovered in 2004 by Novoselov et al. [34], and it has sparked remarkable interest in recent years [35, 36]. Graphene is a good candidate for the adsorption of benzenoid-like compounds because of its static superposition properties and specific large surface area. Zhang et al. described a new fabrication strategy for graphene-coated solid-phase microextraction fibres, which were utilized to enrich polycyclic aromatic hydrocarbons (PAHs) [37]. Wang and coworkers prepared a magnetic microsphere-confined graphene adsorbent, which was utilized in the extraction of PAHs from environmental water samples [38]. Zhao's group prepared graphene-coated magnetic NPS, which were utilized to extract triazine herbicides [23]. The solid-phase microextraction of BMZs by graphene oxide (GO)-bonded, fused-silica fibres has been reported [39]. Luo and coworkers synthesized a poly(ethylene glycol dimethacrylate)-graphene composite, which was used as stir rod sorptive extraction for the extraction of 16 BMZs [40]; good extraction performance was achieved quickly and simply.

In this present study, magnetic graphene was synthesized by in situ chemical precipitation and was used as an adsorbent for the first time. Moreover, a novel analytical methodology based on G-Fe_3_O_4_ MSPE coupled to HPLC was developed for the trace analysis of five BMZs, namely, oxfendazole, mebendazole, flubendazole, albendazole, and fenbendazole in chicken, chicken blood, and chicken liver samples.

## 2. Experimental

### 2.1. Chemicals and Materials

Graphite powder (50 mesh) was purchased from Boaixin Chemical Reagents (Baoding, China). Potassium permanganate (KMnO_4_), concentrated sulphuric acid (H_2_SO_4_), hydrogen peroxide (H_2_O_2_), hydrochloric acid (HCl), nitric acid (HNO_3_), sodium nitrate (NaNO_3_), all phosphate compounds, and all ammonium compounds were purchased from Tianjin Tianda Chemical Reagent Co. (Tianjin, China). Oxfendazole (OXF), mebendazole (MEB), flubendazole (FLU), albendazole (ALB), and fenbendazole (FEN) were purchased from Sigma (Sigma-Aldrich, USA). Stock standard solutions of BMZs in dimethyl sulfoxide/methanol (1 : 9, v/v) (400 mg/L) were prepared and were subsequently diluted. HPLC-grade acetonitrile was obtained from Tedia (Fairfield, OH, USA). Ultrapure water used throughout the experiments was obtained from a Milli-Q gradient A10 system (Millipore, UK). All solutions were filtered through a 0.45-*μ*m filter before being injected into the LC system. All other reagents were of analytical grade.

### 2.2. Instruments

X-ray diffractometry (XRD) was carried out using a Rigaku diffractometer. The size and morphology of the G-Fe_3_O_4_ were determined by scanning electron microscopy (SEM), which was conducted using a 4300 SEM instrument (HITACHI, Japan); Transmission electron microscopy (TEM) was carried out using a Philips Tecnai 10 TEM instrument (Philips, Netherlands). Fourier transform infrared (FT-IR) spectroscopy was carried out on a Nicolet Avatar 330. The magnetic properties were characterized using a Squid-based magnetometer from Quantum Design (San Diego, CA). Centrifugation during sample preparation was performed in a TGL-20LM-B centrifuge equipped with angular rotor (12 × 2.0 mL) (Hunan Star Science Instruments Co. Ltd., China). A QL-901 Vortex (Kylin-bell Lab Instruments Co., Ltd., China) was used for preparing the samples. An Agilent 1200 series LC system equipped with a quaternary pump, autosampler, and VWD ultraviolet detector controlled by Chemstation software was used in all analyses.

### 2.3. Synthesis of Functional Graphene

GO was prepared from natural graphite powder, using a previously reported procedure [41, 42] with slight modifications; in brief, graphite powder was treated with a mixture of concentrated H_2_SO_4_, P_2_O_5_, and K_2_S_2_O_8_ at 80°C. Then, 115 mL of H_2_SO_4_ was added, and the mixture was cooled via immersion in an ice bath with stirring. Next, 5.0 g of NaNO_3_ was added to the mixture_._ Then, preoxidized graphite was added under vigorous stirring to avoid agglomeration. After the graphite powder was well dispersed, 15 g of KMnO_4_ was added slowly under stirring and the temperature was kept below 10°C. The mixture was stirred at room temperature for 2 h. As the reaction progressed, the mixture gradually started to become like a paste and the colour turned light brownish. Next, 230 mL of H_2_O was added slowly to the flask. The addition of water was performed in an ice bath to keep the temperature at 96°C for 1 h. Then 30 mL of 30% H_2_O_2_ was added to terminate the reaction. The resulting solution was allowed to stand overnight, centrifuged, and washed with a solution of HCl followed by water to remove metal ions until the pH was 7; the obtained brown dispersion was centrifuged at 3000 rpm to remove any unexfoliated graphite oxide. Subsequently, it was dried to generate a brown solid, GO.

G-Fe_3_O_4_ was synthesized by the in situ chemical coprecipitation of Fe^3+^ and Fe^2+^ in an alkaline solution in the presence of GO. A solution of NaOH/diethylene glycol (DEG) (10 mg/mL) was prepared by adding 2.0 g sodium hydroxide to 200 mL of DEG; the mixture was refluxed for 1 h at 120°C under a N_2_ atmosphere and then cooled to 70°C. The magnetic composite was prepared by suspending 0.4 g GO in 250 mL of DEG, the mixture was ultrasonicated for 2 h and added to 1.6 g ferric chloride with stirring for 1 h at room temperature. Then, before rapidly injecting 67 mL of the NaOH/DEG solution, it was heated at 220°C within 30 min. After the product was cooled to room temperature, the precipitate was isolated using a magnetic field, and the supernatant was separated from the precipitate by decantation. The impurities in G-Fe_3_O_4_ were removed by washing with water. G-Fe_3_O_4_ was then washed with absolute alcohol until the green yellow colour disappeared. Subsequently, the composite was dried at 80°C for 24 h under vacuum.

### 2.4. Sample Preparation

The chicks were fed using 5 mg/Kg of BMZs in the corn for seven days. Then, they were slaughtered three days later. The thoroughly homogenized chicken, chicken blood, and chicken liver samples were prepared as follows: 5.0 g of the samples, 20 mL of ethyl acetate, 0.30 mL of a 25 g/100 mL of KOH solution, and 0.50 mL of a 1 g/100 mL butylated hydroxytoluene solution were mixed in a 50 mL Eppendorf vial. After the solution was sonicated for 5 min, 0.50 g Na_2_SO_4_ was added and was subjected to centrifugation at 16000 rpm. The resulting clear solution was placed in a 100 mL pear-shaped bottle. Next, the same solution was used to wash the homogenizer and the solution was thoroughly vortexed at room temperature for 2 min, sonicated, and subjected to centrifugation again. Subsequently, the clear solution was added to the aforementioned pear-shaped bottle and dried at 40°C by a rotary evaporator. The residue was immediately dissolved in 10 mL of acetonitrile via ultrasonication, and 10 mL n-hexane was added. The acetonitrile solution was collected and dried via distillation under reduced pressure. Then, the residue was dissolved in 15.0 mL of water for the G-Fe_3_O_4_ sorptive extraction. The concentrations of BMZs in the spiked sample solutions were 0.80 ng/g and 8.0 ng/g.

### 2.5. MSPE Procedure

The MSPE procedure consisted of extraction, desorption, and HPLC analysis. First, 15.0 mg of G-Fe_3_O_4_ was rinsed with acetone and water and dispersed in 15.0 mL of the BMZ water sample solution. Secondly, the mixture was shaken for 30 min to extract the analytes. Subsequently, G-Fe_3_O_4_ was isolated from the solution using a magnet placed at the bottom of the beaker; then, the supernatant was poured off. In order to completely remove the residual solution with a pipette, the particles were moved with a magnet, which was placed on the outside of the bottle wall. The isolated particles were then vortexed with 1.0 mL of acetic acid and methanol (1 : 99, v/v) for 25 min to desorb the analytes. Afterwards, the magnet was placed on the bottom of the bottle and the desorption solution was collected with a micropipette and was subsequently dried with N_2_, to redissolve with 400 *μ*L. Finally, 20.0 *μ*L was injected into the HPLC system for analysis. Prior to the next use, G-Fe_3_O_4_ was washed twice with 5 mL of acetic acid and methanol (1 : 99, v/v) and 5 mL of acetone.

### 2.6. Chromatographic Operating Conditions

All chromatographic separation was performed on a Diamosil C_18_ (250 × 4.6 mm i.d., 5 *μ*m) column from Dikma (Beijing, China). The mobile phase used was acetonitrile/water (pH 3.0 adjusted with 25 mmol/L of ammonium acetate and acetic acid). The acetonitrile phase was increased from 30% to 70% during 0–20 min. The flow rate of the mobile phase was 1.000 mL/min, and the wavelength used for ultraviolet detection was 295 nm.

## 3. Results and Discussion

### 3.1. Morphological Structure of G-Fe_3_O_4_

**Figure 1:**
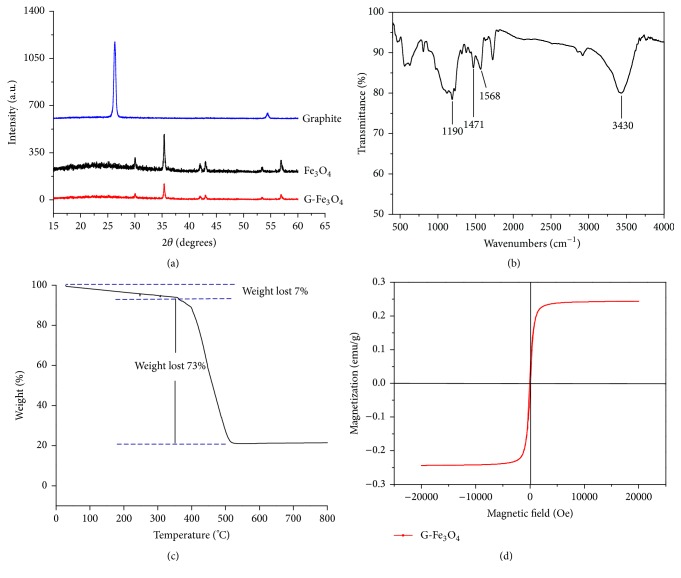
(a) X-ray diffraction (XRD) of graphite, Fe_3_O_4_, and G-Fe_3_O_4_, (b) FT-IR spectra of G-Fe_3_O_4_, (c) thermogravimetric analysis (TGA) of G-Fe_3_O_4_, and (d) VSM magnetization curves of G-Fe_3_O_4_.

X-ray diffraction (XRD) measurements were employed to investigate the structure of the synthesized samples; the graphite and G-Fe_3_O_4_ patterns are shown in Figure 1(a). For graphite, the presence of the characteristic diffraction peak at ca. 26.5°, after the synthesis of G-Fe_3_O_4_, due to G-Fe_3_O_4_ was reduced to graphite and restored to the ordered crystal structure; the characteristic diffraction peak at ca. 26.5° of graphite disappeared. This confirmed the successful oxidation of graphite [43]. Other diffraction peaks of G-Fe_3_O_4_ appeared at 2*θ* = 30.1°, 35.5°, 43.1°, 53.5°, 57.0, and 62.6°, which corresponded to crystal indexes of (2  2  0), (3  1  1), (4  0  0), (4  2  2), (5  1  1), and (4  4  0) of crystalline magnetite (Fe_3_O_4_) [44]. G-Fe_3_O_4_ displayed a similar pattern as that of crystalline magnetite (Fe_3_O_4_).

The FT-IR spectra illustrated in Figure 1(b) revealed the chemical characteristic of the material. For G-Fe_3_O_4_, stretching peak at 3430 cm^−1^ and the peak at 1471 cm^−1^ show the presence of O-H groups. Characteristic stretching peaks of the epoxide functionality were observed at around 1190 cm^−1^, while that of the C=O group was observed at 1568 cm^−1^. Overall, the FT-IR data confirmed that the reaction was successful.

The thermal stability of G-Fe_3_O_4_ was investigated by thermogravimetric analysis (TGA). As shown in Figure 1(c), the curve revealed a weight loss event at 355°C, prior to this temperature, the compound was stable. When the temperature was greater than 355°C, the coordination structures and inorganic components decomposed the Fe_3_O_4_ graphene related to the collapse of the coordination structures and the decomposition of the inorganic components. However, since G-Fe_3_O_4_ was synthesized at 220°C, the compound exhibited high stability and could be obtained in a good yield.

G-Fe_3_O_4_ should possess sufficient magnetic properties to allow for rapid separation under a magnetic field. The VSM magnetization curves of G-Fe_3_O_4_ at 25°C are shown in Figure 1(d); they indicated that G-Fe_3_O_4_ exhibited excellent superparamagnetic behaviour. The saturation magnetization intensity of G-Fe_3_O_4_ was 0.26 emu g^−1^. Therefore, G-Fe_3_O_4_ can be used for magnetic separation from solution by using a strong magnet.

**Figure 2:**
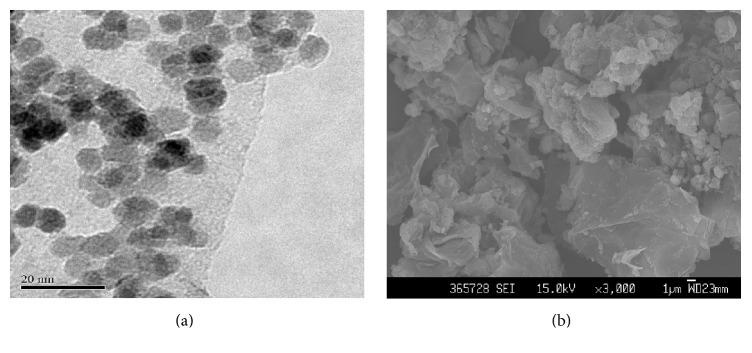
(a) TEM and (b) SEM images of G-Fe_3_O_4_ composites.

The TEM and SEM images of the G-Fe_3_O_4_ composite are shown in Figure 2 and they illustrate the characteristic features of single-layer G sheets. It can be clearly seen that the carbon sheets resembled crumpled silk waves (Figure 2(b)). As shown in Figure 2(a), the iron oxide NPS were successfully coated on the surface of G to form the G-Fe_3_O_4_ composite. The average size of the Fe_3_O_4_ NPS was about 5 nm, as estimated by TEM. Moreover, the Fe_3_O_4_ NPS were well distributed on the graphene sheets, which were nearly flat and comprised a large area of up to several square micrometres. Because the loading degree was near saturation, some Fe_3_O_4_ NPS were slightly aggregated.

### 3.2. Effect of Extraction Conditions on Extraction Efficiency

The extract was based on *π*-*π* interactions between the G-Fe_3_O_4_ and the BMZs. In order to determine the optimal extraction conditions, water spiked with an appropriate amount of BMZs was used to study the extraction performance under different experimental conditions. Several parameters, including the amount of G-Fe_3_O_4_, extraction solvent, extraction time, and desorption conditions, were explored in order to achieve the best extraction efficiency. All experiments were performed in triplicate and the means of the results were reported.

#### 3.2.1. Extraction Conditions

**Figure 3:**
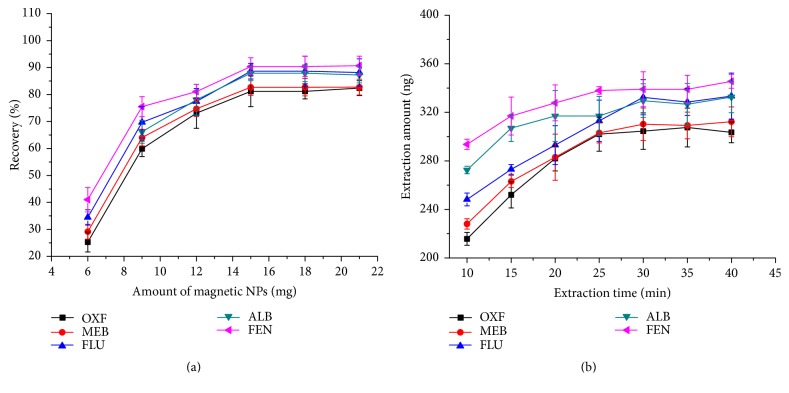
Effect of experimental conditions on the extraction efficiency. (a) Effect of the amount of sorbent and (b) effect of extraction time.

In order to determine the optimum amount of adsorbent (G-Fe_3_O_4_) for the extraction of BMZs (OXF, MEB, FLU, ALB, and FEN), the dosages of G-Fe_3_O_4_ were varied from 6.0 to 21.0 mg. As shown in Figure 3(a), the maximum extraction efficiency was obtained with 15.0 mg of G-Fe_3_O_4_. When the amount of adsorbent was greater than 15.0 mg, the recovery was unchanged. Accordingly, 15.0 mg G-Fe_3_O_4_ was selected as the optimal amount of adsorbent. Generally, in MSPE, sufficient contact time is required for the analytes to attain adsorption equilibrium on the sorbent. In order to elucidate the effects of extraction time on the adsorption efficiency of BMZs, the extraction time was varied from 10 to 40 min, while other parameters were held constant; the results are shown in Figure 3(b). When the sample solutions were shaken for 30 min, the extraction amounts of all BMZs were the greatest and remained almost content for extraction times greater than 30 min; prolonged extraction times did not increase the extraction amount of the analytes significantly, indicating that the extraction equilibrium could be achieved in a short amount of time. Hence, an extraction time of 30 min was selected.

#### 3.2.2. Desorption Conditions

**Figure 4:**
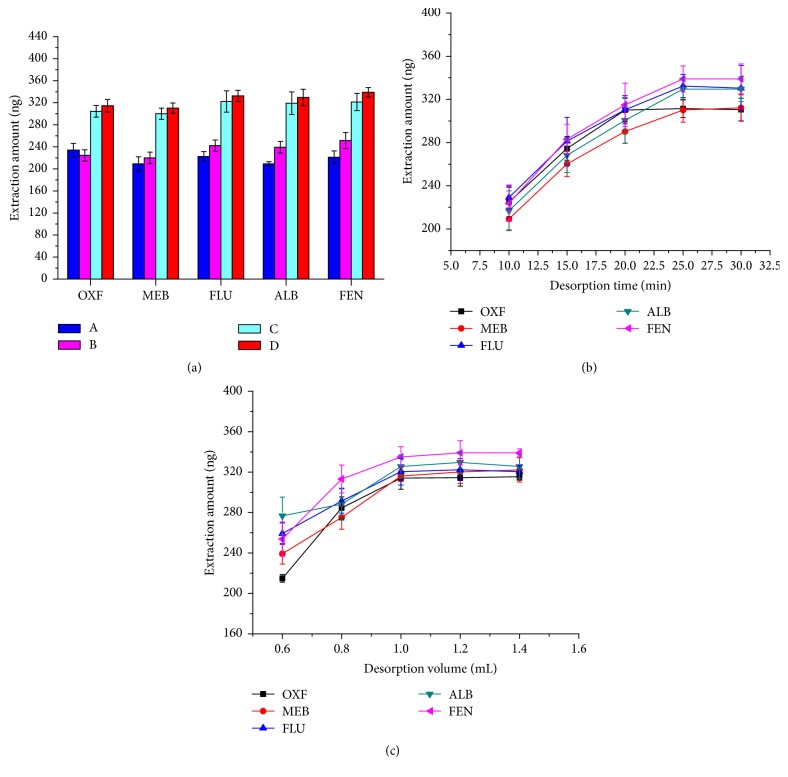
Effects of experimental conditions on the desorption efficiency. (a) Effects of desorption solvent, A: acetonitrile; B: methanol; C: acetic acid/acetonitrile (1 : 99, v/v); D: acetic acid/methanol (1 : 99, v/v); (b) desorption time; and (c) volume of desorption solvent.

The analytes should be completely desorbed from the G-Fe_3_O_4_ particles prior to HPLC-UV analysis. The desorption of BMZs required that the *π*-*π* interactions be disrupted, so polar solvents were selected as the desorption solvents. In this work, the desorption of BMZs from G-Fe_3_O_4_ was studied using different organic solvents, including acetonitrile, methanol, and mixtures of acetic acid and acetonitrile (1 : 99, v/v) and acetic acid and methanol (1 : 99, v/v). The results shown in Figure 4(a) reveal that, under the same extraction and elution conditions, the desorption power of acetic acid/methanol (1 : 99, v/v) and acetic acid/acetonitrile (1 : 99, v/v) was much stronger than either methanol or acetonitrile; thus, matrix interferences were serious when using acetic acid/acetonitrile (1 : 99, v/v) solvent. Hence, it was selected as the desorption solvent. In addition, the influence of desorption time (from 10 to 30 min) on the desorption efficiency of BMZs was investigated. In general, longer desorption times led to better extraction efficiencies, shown in Figure 4(b), and adsorption equilibrium was achieved after about 25 min. Therefore, 25 min was ultimately chosen as the preferred desorption time. Under the optimized experimental conditions, the adsorbed analytes were desorbed with 0.60–1.40 mL acetic acid and methanol (1 : 99, v/v) by vortexing for 25 min (Figure 4(c)). When 1.00 mL of the solvent was used, the analytes were almost completely desorbed. Therefore, 1.00 mL of a mixture of acetic acid and methanol (1 : 99, v/v) was used as the desorption solvent.

### 3.3. Adsorption Performance of BMZs by G-Fe_3_O_4_

#### 3.3.1. Determination of G-Fe_3_O_4_ Adsorption Capacity

**Figure 5:**
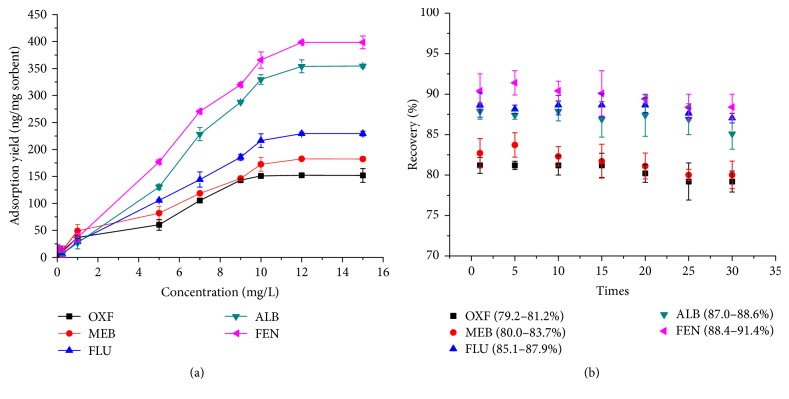
(a) Adsorption capacity of G-Fe_3_O_4_ and (b) reusability of G-Fe_3_O_4_ in the extraction of BMZs.

In this study, adsorption capacity was defined as the maximum amount of BMZs extracted by G-Fe_3_O_4_. The G-Fe_3_O_4_ sorbent was characterized in terms of capacity, which was directly related to the amount of graphene. The extraction capacity of the G-Fe_3_O_4_ sorbent was determined by exposing the sorbent to water solutions containing increasing amounts of BMZs (0.050–15.0 mg/L) for 30 min. The results are shown in Figure 5(a). The G-Fe_3_O_4_ sorbent was able to extract up to 12 mg/L BMZs, and the amount of extracted BMZs reached a plateau at higher concentrations. The sorbent capacities were 152, 183, 229, 354, and 399 ng BMZs/mg G-Fe_3_O_4_ sorbent, respectively. The enrichment factors were measured as 114–299 for the selected five BMZs, indicating the remarkable preconcentration ability.

#### 3.3.2. Reusability of the Sorbent

In order to investigate the reusability of the G-Fe_3_O_4_ sorbent, it was washed twice with 5 mL of acetic acid and methanol (1 : 99, v/v) and 5 mL of acetone before it was reused in subsequent MSPE. The experimental results (Figure 5(b)) revealed that the G-Fe_3_O_4_ sorbent could be reused at least 30 times without any significant loss of sorption capacity, and satisfied recovery with RSD (*n* = 5) lower than 9.6% was obtained on the extraction of five selected BMZs. This indicated that the iron oxide NPS were successfully coated on the surface of graphene in the G-Fe_3_O_4_ composite, thus ensuring suitable stability during reuse.

### 3.4. Analytical Performance of MSPE

Under the optimized conditions, some analytical performance parameters of the method, including linear range (LR), correlation coefficient (*R*^2^), limit of detection (LOD), and limit of quantification (LOQ), were investigated.

#### 3.4.1. Analytical Figures of Merit

A series of working solutions containing OXF, MEB, FLU, ALB, and FEN at concentrations ranging from 0.100 to 100 *μ*g/L were prepared in order to establish the calibration curve. For each concentration, seven replicate extractions and determinations were performed under the optimized experimental conditions. The calibration data are listed in Table 1. Good linearity was observed over the concentration range of 0.100–100 *μ*g/L for OXF, MEB, FLU, ALB, and FEN. The correlation coefficients ranged from 0.9966 to 0.9998. The limits of detection (LODs) were within the range 17.2–32.3 ng/L (LODs were estimated on the basis of 3 : 1 signal to noise ratios). The resultant repeatability, expressed as relative standard deviation (RSD), varied from 3.4% to 7.6%. The results indicated that the method was highly sensitive and reproducible.

#### 3.4.2. Application of BMZs Analysis in Real Samples

**Figure 6:**
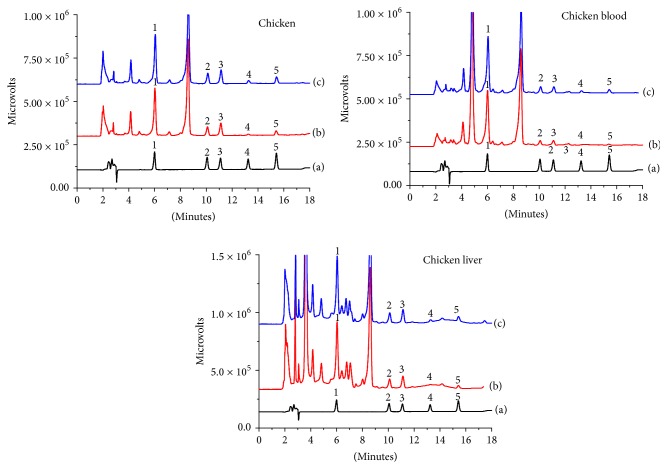
Chromatograms of BMZs in chicken, chicken blood, and chicken liver samples. (a) the standard solution at 1.0 mg/L, (b) a sample solution extracted by G-Fe_3_O_4_ MSPE, and (c) 0.8 ng/g of the spiked sample solution extracted by G-Fe_3_O_4_ MSPE. The peaks corresponded to the following BMZs: 1. OXF, 2. MEB, 3. FLU, 4. ALB, and 5. FEN.

The MSPE-HPLC method developed in this study was used to analyze several food samples, including chicken, chicken blood, and chicken liver samples. The MSPE showed maximal elimination of the matrix interferences and enhancement of the sensitivity. The amounts of OXF, MEB, FLU, and FEN in these samples ranged within 13.0–20.2, 1.62–4.64, 1.94–6.42, and 0.292–1.04 ng/g, respectively. ALB was also detected in some samples; the results are shown in Table 2. To estimate the influence of the matrix all of the samples were spiked with different concentrations (Table 2). The satisfactory recoveries of the method ranged from 83.0% to 115% with RSDs between 2.7% and 7.9% for chicken, chicken blood, and chicken liver samples, which suggested that the method was suitable for the analysis of the BMZs in complex samples. Typical chromatograms of BMZs from the samples are shown in Figure 6.

## 4. Conclusion

In the present study, G-Fe_3_O_4_ was facilely synthesized by in situ chemical coprecipitation and it exhibited a great adsorption ability and good stability in the MSPE of BMZs. The proposed method for the determination of BMZs in chicken, chicken blood, and chicken liver samples was established by combining G-Fe_3_O_4_ as an effective adsorbent with HPLC-UV. The LODs were in the range of 17.2–32.3 ng/L, and the recoveries of the method ranged between 83.0% and 115%; the RSDs were less than 7.9%. Furthermore, G-Fe_3_O_4_ could be reused at least 30 times without a significant loss in the sorption capacity or magnetism. Moreover, the G-Fe_3_O_4_ exhibited a remarkable preconcentration ability for five BMZs, and satisfactory repeatability and recoveries were obtained. The use of G-Fe_3_O_4_ was also faster and less expensive than other previously reported methods. The developed method serves as a simple and highly efficient extraction and preconcentration technique for BMZs in chicken, chicken blood, and chicken liver samples. MSPE based on the G-Fe_3_O_4_ can also be used for the enrichment of other trace organic pollutants in food samples.

## Figures and Tables

**Table 1:** Analytical performance and results for HPLC-UV determination of five BMZs using magnetic graphene.

Compounds	Equation of linearity	*R* ^2^	Range	LOD^a^	LOQ^a^	RSD^b^
			(*µ*g/L)	(ng/L)	(ng/L)	(%) (*n* = 7)
OXF	*Y* = 9.31 × 10^4^*X* − 6.4 × 10^2^	0.9966	0.100–100	19.4	58.7	3.4
MEB	*Y* = 7.62 × 10^4^*X* − 9.6 × 10^2^	0.9992	0.100–100	28.3	84.6	5.7
FLU	*Y* = 7.26 × 10^4^*X* − 7.9 × 10^2^	0.9998	0.100–100	27.4	82.8	7.6
ALB	*Y* = 6.45 × 10^4^*X* − 1.1 × 10^3^	0.9988	0.100–100	32.3	97.4	5.4
FEN	*Y* = 1.01 × 10^5^*X* − 8.4 × 10^2^	0.9986	0.100–100	17.2	52.2	4.9

^a^LOD and LOQ were estimated on the basis of 3 : 1 and 10 : 1 signal to noise ratios, respectively.

^b^The method precision was monitored with 1.0 *µ*g/L mixed standard solution and the RSD of extraction amounts of five BMZs.

**Table 2:** Analysis of BMZs in food samples using the magnetic solid-phase extraction coupled to HPLC (*n* = 5).

Samples	Analytes	Original amount (ng/g)	RSD (%)	Spiked concentration (ng/g)			
				0.80 ng/g		8.0 ng/g	
				Recovery (%)	RSD (%)	Recovery (%)	RSD (%)
Chicken	OXF	13.0	3.9	84.1	4.2	94.8	3.4
	MEB	3.00	7.2	83.0	4.9	105	3.4
	FLU	4.56	6.2	84.8	5.9	105	5.1
	ALB	0.450	6.3	89.6	5.8	107	4.8
	FEN	1.04	8.7	84.7	7.9	92.4	6.4

Chicken blood	OXF	17.5	3.7	93.0	3.9	93.6	4.2
	MEB	1.62	5.1	95.7	3.0	101	2.9
	FLU	1.94	6.4	102	4.7	102	4.1
	ALB	N.Q.	—	95.7	4.3	112	3.5
	FEN	0.292	8.9	87.2	6.0	94.6	4.5

Chicken liver	OXF	20.2	3.5	85.9	4.2	90.9	3.5
	MEB	4.64	5.6	88.7	5.1	115	4.3
	FLU	6.42	4.3	91.8	3.4	108	2.7
	ALB	0.342	5.7	97.8	3.9	112	3.4
	FEN	1.01	6.9	95.0	4.0	97.3	3.6

*Note*. N.Q.: not quantified.
